# Cost-Effectiveness of Hepatitis C Treatment for People Who Inject Drugs and the Impact of the Type of Epidemic; Extrapolating from Amsterdam, the Netherlands

**DOI:** 10.1371/journal.pone.0163488

**Published:** 2016-10-06

**Authors:** Daniëla K. van Santen, Anneke S. de Vos, Amy Matser, Sophie B. Willemse, Karen Lindenburg, Mirjam E. E. Kretzschmar, Maria Prins, G. Ardine de Wit

**Affiliations:** 1 Department of Infectious Disease Research and Prevention, Public Health Service of Amsterdam, Amsterdam, the Netherlands; 2 Julius Center for Health Sciences and Primary Care, University Medical Center Utrecht, Utrecht, the Netherlands; 3 Department of Gastroenterology and Hepatology, Academic Medical Center, Amsterdam, the Netherlands; 4 National Institute of Public Health and the Environment, Bilthoven, the Netherlands; 5 Department of Infectious Diseases, Center for Infection and Immunology Amsterdam (CINIMA), Academic Medical Center (AMC), Amsterdam, the Netherlands; Kaohsiung Medical University Chung Ho Memorial Hospital, TAIWAN

## Abstract

**Background:**

People who inject drugs (PWID) are disproportionally affected by the hepatitis C virus (HCV) infection. The efficacy of HCV treatment has significantly improved in recent years with the introduction of direct-acting antivirals (DAAs). However, DAAs are more costly than pegylated-interferon and ribavirin (PegIFN/RBV). We aimed to assess the cost-effectiveness of four HCV treatment strategies among PWID and treatment scale-up.

**Methods:**

An individual-based model was used describing HIV and HCV transmission and disease progression among PWID. We considered two epidemiological situations. A declining epidemic, based on the situation in Amsterdam, the Netherlands, and a stable HCV epidemic, as observed in other settings. Data on HCV incidence, prevalence, treatment setting and uptake were derived from observed data among PWID in Amsterdam. We assessed the incremental cost-effectiveness ratio (ICER, costs in €/quality-adjusted life year (QALY)) of four treatment strategies: 1) PegIFN/RBV; 2) sofosbuvir/RBV for genotype 2–3 and dual DAA for genotype 1–4; 3) Dual DAA for all genotypes; 4) Dual DAA with 3x treatment uptake.

**Results:**

In both types of epidemic, dual DAA therapy was most cost-effective strategy. In the declining epidemic, dual DAA yielded an ICER of 344 €/QALY while in the stable epidemic dual DAA led to cost-savings. Scaling-up treatment was also highly cost-effective. Our results were robust over a range of sensitivity analyses.

**Conclusion:**

HCV treatment with DAA-containing regimens is a highly cost-effective intervention among PWID. Based on the economic and population benefits of scaling-up treatment, stronger efforts are needed to achieve higher uptake rates among PWID.

## Introduction

People who inject drugs (PWID) are disproportionally affected by the hepatitis C virus (HCV) infection [1] as sharing unsafe injecting equipment poses a high risk for HCV transmission [2]. The global anti-HCV prevalence among PWID is estimated to be 67% [3], though the HCV epidemic among PWID differs by geographical region. In Eastern European countries such as Poland, [4] a continued spread of HCV has been documented among PWID whereas a declining HCV epidemic has been observed in Western European countries such as the Netherlands [5]. HCV infection is one of the leading causes of liver-related disease and cirrhosis usually develops around 20 to 30 years after infection in 16% to 41% of HCV-infected individuals [6]. A modeling study extrapolating from the Amsterdam Cohort Study (ACS) among PWID reported that the burden of HCV infection is expected to rise in in the next decade in the absence of HCV treatment and/or treatment scale-up [7].

Treatment with pegylated-interferon and ribavirin (PegINF/RBV) became available after 2001 with an overall sustained virological response (SVR) ranging from 33–79% [8]. Among PWID, similar SVR have been documented [9] and PegINF/RBV has been shown to be cost-effective among current and former PWID [10]. However, PegIFN is known to cause many side effects and the burdensome treatment may take up to 48 weeks [9]. Furthermore, as active drug used to be a contraindication for HCV treatment [11], and other barriers, such as limited access to care and lack of a social support system among PWID [12], a relatively low HCV-treatment uptake among PWID has been reported [13]. Additionally, especially with this type of HCV treatment, PWID require a flexible and permissive setting with extensive follow-up and extra healthcare support due to psychiatric comorbidities and the lifestyle of PWID [14].

New all-oral treatment regimens with direct-acting antivirals (DAAs) are highly effective, with SVR rates reported up to 95–100% [15]. A cost-effectiveness study among PWID in Australia showed that DAA treatment can be cost-effective [16]. In the Netherlands, DAAs are reimbursed for all HCV-infected individuals, irrespective of their fibrosis stage, since November 2015 [17]. However, despite the availability of DAAs in the Netherlands, the high costs of DAAs and the lifestyle of PWID may still pose barriers to provide treatment to PWID.

Beyond the costs, the cost-effectiveness of HCV treatment may depend on several factors such as HCV-screening uptake and whether PWID with a re-infection after successful HCV treatment are re-treated. The type of HCV epidemic may also influence the cost-effectiveness. In a stable epidemic, with a stable PWID population inflow and HCV incidence, transmission is on-going and treatment may prevent new infections (treatment as prevention). In contrast, in a declining epidemic as observed among PWID in the Netherlands [5], small population prevention effects can be expected, thus limiting the impact of treatment to the treated population only. To date, few studies have assessed the cost-effectiveness of DAAs among PWID and whether the cost-effectiveness of HCV treatment depends on the type of HCV epidemic. In this study we aim to assess the cost-effectiveness of four HCV treatment strategies among PWID and HCV-treatment scale-up. Furthermore, we aim to explore the impact of the type of epidemic on the cost-effectiveness of DAAs and on the chronic HCV prevalence over time.

## Methods

### Model

An individual-based model describing demographic changes and infection dynamics of HIV and HCV was employed. This model was used previously to study the effects of harm reduction policy on the spread of HIV and HCV in Amsterdam [18], as well as the potential of treatment as prevention for HIV among PWID [19]. Since detailed features have been described before [18],below we describe only the main features of this model and how it was adapted. In summary, PWID entered the model at the beginning of their injecting career; subsequently they could cease injecting, relapse, acquire HCV and/or HIV or die and leave the model. Cycle length in the model was one month. PWID population inflow was based on back calculations from the number of participants in methadone programs in Amsterdam [7]. The probability of PWID participants having acquired HIV or HCV depended on the syringe-sharing rate and the probability that a borrowed syringe came from an infected PWID. PWID who cleared an HCV infection, either spontaneously or after successful treatment, were at risk of re-infection. For the purpose of this study, the model was expanded with information on HCV genotype (grouped into: genotype 1–4 (G1-4) and genotype 2–3 (G2-3)) (Text A in S1 File), HCV disease progression, HCV screening, and HCV treatment. Based on data from the Amsterdam Cohort Study, 70% of HCV-positive PWID were infected with G1-4 [20].

We considered two epidemiological scenarios. For the first epidemiological scenario, the declining HCV epidemic, demographic parameters were estimated from the ACS [21]. In Amsterdam, HCV prevalence was 60% during 2006–2012 among ACS participants [22]. The number of PWID and HCV and HIV incidence have declined over time [5, 7]. HCV and HIV incidence were estimated to be 27.5 and 8.5 per 100 person years in the late 1980s, respectively, and declined to almost 0 for both infections in 2013 [5].

As the Dutch epidemiological situation is not representative for the majority of countries, we made a counter-factual scenario to explore the impact of the type of epidemic on the cost-effectiveness of DAAs in a more general setting. The model demographics were adapted to obtain a stable HCV and HIV epidemic. The number of new PWID entering the population was set at 4 per month; resulting in a stable population size of approximately 1,500 PWID (this number is comparable to the peak of the Amsterdam PWID population size as in 1985). Risk behavior of PWID in this scenario was similar to that at the start of the Amsterdam epidemic. In contrast to the declining epidemic, syringe sharing frequency did not decline over time. Prior to the introduction of HCV treatment, we ran the model until all variables reached their equilibrium distributions.

### Base case parameters

#### Natural history of HCV

**Fig 1:**
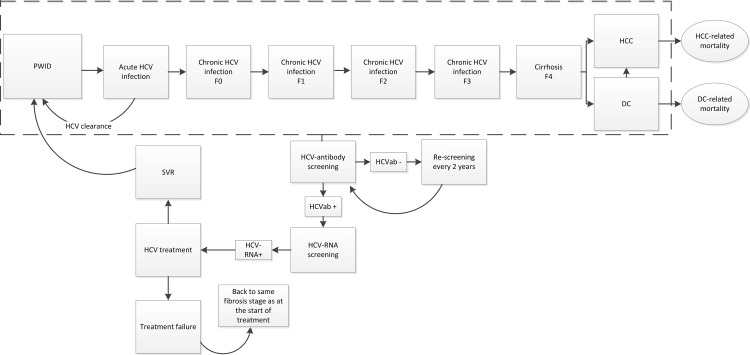
Natural history of HCV, screening, and treatment among PWID ^a^, ^a^ PWID enter the model uninfected with HCV and HIV and may follow different health state trajectories as shown in the flow diagram. At any point, PWID may acquire HIV or exit the model due to background mortality or HIV-related mortality among HIV-infected PWID. Dashed lines depict the health states where PWID can be screened for HCV. Arrowed lines depict annual transition probabilities with the exception of HCV-antibody screening (which takes place every two years).

In Fig 1, a schematic overview of the model is given. The model simulates PWID through each fibrosis stage (F0-F4) by age group, HIV status, and sex (Table 1 and Text B in S1 File). HIV/HCV-coinfected PWID had a two-fold risk to progress to the following fibrosis stage [23] and had a lower spontaneous clearance rate than HCV-monoinfected PWID [24,25]. Cirrhotic individuals could develop decompensated cirrhosis (DC) or hepatocellular carcinoma (HCC) and consequently, die as a result of liver-related causes. Liver transplantation was not incorporated in the model as active alcohol and drug use is a contra-indication [26]. Age-dependent all-cause mortality among HIV-uninfected PWID was based on data from the ACS [18]. Mortality estimates for HIV-infected PWID were based on data from the CASCADE Collaboration among HIV seroconverters [27].

**Table 1:** Base case demographics, annual transition probabilities, and SVR probabilities per treatment scenario.

Demographics	%		Source
**Sex distribution**			[22]
Men	0.64		
**Genotype distribution**			[28]
1–4	0.70		
2–3	0.30		
**Annual transition probabilities**			
	**HIV-negative**	**HIV-positive**	
**Acute HCV to chronic HCV**			[25,29,41]
Women	0.58	0.64 ^a^	
Men	0.80	0.89 ^a^	
**Fibrosis progression in METAVIR staging** (yearly transition is a METAVIR transition) ^b^			
**Women**			[30, 31]
<49	0.05	0.10	
50 to 59	0.12	0.25	
60 to 69	0.22	0.44	
> = 70	0.30	0.60	
**Men**			[30, 31]
<49	0.03	0.06	
50 to 59	0.07	0.13	
60 to 69	0.11	0.23	
70–79	0.15	0.31	
> = 80	0.21	0.42	
**Disease progression after cirrhosis (F4)**			
**To DC or HCC** ^**c**^			
F4 to DC	0.039	0.059	[32,42]
DC to HCC	0.068	0.102	[33,42]
F4 to HCC	0.021	0.032	[30,42]
**Death**			[30]
DC to death	0.31	^d^	
HCC to death	0.43	^d^	
**Treatment scenarios–SVR probabilities**			
	**HIV-negative**	**HIV-positive**	
**Scenario 1: PegIFN/RBV**			
Genotype 1–4 (48 weeks)			[34, 35]
F0-F2	0.47	0.28	
F3-F4	0.33	0.20	
Genotype 2–3 (24 weeks)			[35, 36]
F0-F2	0.76	0.71	
F3-F4	0.52	0.47	
**Scenario 2: DAA/RBV & Dual DAA**				
***Dual DAA therapy***			[15, 37]
Genotype 1–4 (12 weeks)			
F0-F4	0.95	0.95	
***DAA/RBV***			[38–40]
Genotype 2–3 (22 weeks ^e^^,^^f^)			
F0-F4	0.90	0.90	
**Scenario 3&4: Dual DAA therapy**			[15, 37]
All genotypes (12 weeks)			
F0-F4	0.95	0.95	

Abbreviations: PegIFN: pegylated-interferon; RBV: Ribivarin; No: Number; SVR: sustained virological response; DAA: direct-acting antiviral

a Clearance rate reported to be 15 and 20% among HIV/HCV-coinfected individuals,; therefore we assumed an overall clearance rate of 17% among them. Clearance rate by sex among HIV/HCV-coinfected was proportional to that among HIV-negative individuals.

b 2 times the fibrosis progression rate of HIV-negative individuals

c 1.5 times the progression rate among HIV-negative individuals

d Not related to HIV status

e We calculated a weighted average for the number of treatment weeks for those with genotype 2–3 as those with genotype 2 should be treated for 12 weeks while those with genotype 3 should be treated for 24 weeks with SOF/RBV. We assumed that a maximum of 20% of PWID [28] in this genotype group are infected with genotype 2, therefore the weighted number of weeks of treatment is 21.6.

f At the time the model was built, data for 24 weeks treatment for genotype 3 was scarce and the Positron trial showed similar SVR among cirrhotic and non-cirrhotic patients with genotype 2. In post-hoc sensitivity analyses SVR probability for F3-F4 was set at 0.70.

### HCV screening

HCV screening in both epidemics was based on the situation in Amsterdam, where PWID attending harm-reduction programs (HRP) have been routinely offered screening for HCV and HIV since 2001. Around 75% of PWID living in Amsterdam received or had received methadone substitution therapy [43] and, based on a pilot study in Amsterdam, around 80% of PWID in HRP were screened for HCV in methadone programs (Epidemiology, Health Promotion and Innovation Department, PHSA-personal communication). PWID who test HCV-antibody negative are screened every two years for HCV antibodies. PWID who test HCV-antibody positive are tested for HCV RNA and HCV genotyping is performed. In the model, we assumed that 20% of the total PWID population would never be screened for HCV reflecting the Amsterdam estimated percentage of PWID that abstain from screening and/or HRP.

### Treatment setting and uptake

In 2005, a special unit to treat HCV-RNA positive PWID with PegIFN/RBV was launched outside a hospital setting in Amsterdam (DUTCH-C project); details of this project have been previously described [9]. In summary, treatment was coordinated by a multidisciplinary team consisting of a physician and a nurse from the Public Health Service of Amsterdam (PHSA), and a hepatologist and a virologist from the Amsterdam Medical Center (AMC). The nurse gave counseling, provided PegINF injections every week, and contacted family members or friends to provide health counseling and/or support. In our analyses, based on this past experience in Amsterdam and expert opinion, 15 PWID (out of an estimated 1,783 HCV-RNA-positive PWID in Amsterdam in 2015), irrespective of their fibrosis stage, were treated at the PHSA annually; corresponding to a 1% treatment uptake rate in 2015 (Text C in S1 File). Chronically infected PWID were assumed to complete the course of treatment. However, SVRs in our study were based on intention to treat analyses where those lost to follow-up or who stopped treatment were included as treatment failures in the SVR calculation. After achieving SVR, PWID could get re-infected. Treatment-experienced PWID were not eligible for re-treatment.

### Treatment strategies

Dutch treatment guidelines currently recommend a combination of two DAAs (dual DAA) for genotype 1 and 4 and either dual DAA therapy or sofosbuvir (SOF)/RBV for genotype 2 and 3 [17]. The following four HCV-treatment strategies for treatment-naïve PWID were evaluated (schematic representation: Table A in S1 File):

1. Treatment with PegIFN/RBV (24 weeks G2-3 and 48 weeks G1-4): standard treatment in the Netherlands until November 2014. This strategy is used as a comparator strategy to calculate the cost-effectiveness of DAAs.
2. SOF/RBV for G2-3 (weighted average 22 weeks) and dual DAA for G1-4 (12 weeks).
3. Dual DAA therapy for all genotypes (12 weeks).
4. Dual DAA therapy for all genotypes with a three times higher treatment uptake.

HCV-treatment uptake in our study is based on PegIFN/RBV treatment among PWID within the DUTCH-C project which was limited by the eligibility for PegIFN/RBV treatment and the manpower available given the long treatment durations. Based on reduced treatment duration and fewer side effects with DAA-containing regimens, we believe that with the same health-service capacity, a higher treatment uptake could be attained. Therefore a strategy with a higher uptake with dual DAAs was also assessed incrementally. SVRs for DAAs are mainly based on clinical trial data as limited real-world results were available (Table 1). We assumed no difference in SVR rates with DAAs by HIV status based on the PHOTON-2 and ALLY-2 trial [37, 44]. The backbone for all DAA treatment regimens is SOF. Dual DAA treatment SVR is based on the high SVR rates observed in trials with sofosbuvir combined with daclatasvir [45, 46] (Table 1 and Text D in S1 File).

### Utilities

Utilities were based on an UK Health Technology Assessment Report [47]. We multiplied all utilities by 0.85 to reflect the lower base-case quality of life among PWID [10]. Treatment with PegIFN/RBV leads to a 0.11 decrement in quality of life [47]. As a recent study [48] suggests a limited impact of DAA regimens on the quality of life, we conservatively assumed half the loss in quality of life during treatment with DAAs compared with PegIFN/RBV (Table 2). As no utilities were available during treatment among cirrhotic patients, we assumed a proportional decrement in utility similar to the difference between mild (F0-F1) and moderate fibrosis (F2-F3) (Table 2).

**Table 2:** Costs and utilities used in the cost-effectiveness analysis.

Costs of treatment			Distribution	Source
**Treatment strategy**		**Costs in euro 2014**		
PegIFN/RBV G1-4 (48 weeks)		29,712	Gamma (k = 29,712, θ = 1)	Own cost calculation ^a^
PegIFN/RBV G2-3 (24 weeks)		19,298	Gamma (k = 19,298, θ = 1)	Own cost calculation ^a^
DAA/RBV G2-3 (22 weeks)		106,476	Gamma (k = 106,476, θ = 1)	Own cost calculation ^a^
Dual DAA therapy ^b^ (12 weeks)		84,216	Gamma (k = 84,216, θ = 1)	Own cost calculation ^a^
**Annual costs per health state before or after treatment**				
Chronic HCV	F0-F2	130	Gamma (k = 130, θ = 1)	AMC/PHSA
	F3	289	Gamma (k = 289, θ = 1)	AMC/PHSA
	F4	433	Gamma (k = 433, θ = 1)	AMC/PHSA
	DC	27,905	Gamma (k = 27,905, θ = 1)	[49]
	HCC	21,389	Gamma (k = 21,389, θ = 1)	[49]
After SVR	F0-F2^c^	179	Gamma (k = 179, θ = 1)	AMC/PHSA
	F3	227	Gamma (k = 227, θ = 1)	AMC/PHSA
	F4	496	Gamma (k = 496, θ = 1)	AMC/PHSA
**Utilities** ^**d**^^,^^**h**^^,^^**i**^				
		**Utility value**		
SVR	F0-F1	0.82	Beta (α = 29.6, β = 12.87)	[47]
	F2-F3	0.72	Beta (α = 38.19, β = 24.21)	[47]
	F4	0.62	Beta (α = 46.77, β = 41.98)	^e^
Chronic HCV	F0-F1	0.77	Beta (α = 33.90, β = 17.89)	[47]
	F2-F3	0.66	Beta (α = 43.34, β = 33.91)	[47]
	F4	0.55	Beta (α = 52.78, β = 60.12)	[47]
DC		0.45	Beta (α = 61.37, β = 99.07)	[47]
HCC		0.45	Beta (α = 61.37, β = 99.07)	[47]
Treatment with PegIFN	F0-F1	0.66	Beta (α = 43.34, β = 33.91)	[47]
	F2-F3	0.55	Beta (α = 52.78, β = 60.12)	[47]
	F4	0.45	Beta (α = 61.37, β = 99.07)	^e^
PegIFN-free treatment	F0-F1	0.72	Beta (α = 38.19, β = 24.21)	^f^
	F2-F3	0.61	Beta (α = 47.63, β = 44.23)	^f^
	F4	0.50	Beta (α = 57.08, β = 77.22)	^f^

Abbreviations: AMC: Amsterdam Medical Center; PHSA: Public Health Service of Amsterdam; G: genotype; SVR: sustained virological response

a For more detailed information, see Table B in S1 File.

b Costs of sofosbuvir and daclastavir in the Netherlands as in 2016.

c Healthcare utilization only once after achieving SVR.

d Utilities were multiplied by 0.85 in the analyses to account for drug dependency.

e Similar utility decrement assumed as the decrement from F0-F1 to F2-F3 in the chronic HCV health state.

f During IFN-free treatment, we assumed a lower utility decrement (-0.05 decrement instead of -0.11 decrement during treatment with PegIFN) than the decrement during PegIFN treatment.

h In order to use the utilities by Shepherd et al. we assumed that F0-F1 = mild disease, F2-F3 = moderate disease, and F4 = severe disease based on expert medical opinion.

i parameters from the beta distribution of the utilities based on the utilities accounted for drug dependency (see d)

### Healthcare utilization and costs

We adopted a healthcare perspective in our study. Healthcare utilization and costs associated with HCV disease state were subdivided based on treatment outcome: no SVR (untreated or unsuccessfully treated) or SVR after treatment (Table 2). Healthcare utilization (e.g., diagnostic tests) before, during, and after treatment were based on Dutch HCV guidelines [50] and standard treatment at the AMC determined by an hepatologist (Table B in S1 File). Healthcare costs were obtained from the Dutch Health Authority, Academic Medical Center (AMC), and the PHSA, and included overhead costs. For DAA-treatment regimens, the number of diagnostic tests and consultations was adapted to reflect the shorter treatment duration. Costs for PegIFN/RBV were based on the mean costs of treatment, including side effects, in the Netherlands [51]. Weekly sofosbuvir and daclatasvir costs in the Netherlands were 3,621 and 2,252 euros, respectively [52]; daclatasvir combined with sofosbuvir costs was used for the dual DAA therapy scenario as both medications are pan-genotypic. It is important to note that the Dutch ministry negotiated prices with pharmaceutical companies and actual DAA costs have not been made public, hence DAA prices might be lower at present. All costs were indexed to 2014 prices. We included specific costs for treating PWID based on the healthcare utilization accrued from the DUTCH-C project, determined in consultation with the medical coordinator (Table 2). As DAA regimens have been shown to have fewer side effects than PegIFN/RBV [15, 37–39, 53, 54], we conservatively assumed the costs of side effects with DAAs to be half of that with PegIFN/RBV (Table A in S1 File). Table 2 displays “treatment costs” which are the sum of medication and healthcare-related cost (e.g., HCV RNA monitoring).

### Analyses

Total costs and effects (quality adjusted life-years (QALYs)) were calculated by adding up all costs and QALYs over a 15-year time horizon (from 2015 onwards), among PWID with a chronic HCV infection and PWID who were screened and treated during the modeled period. The ICER was calculated by dividing the difference in costs between two strategies by the difference in QALYs and represents the incremental cost associated with an additional QALY gained following a strategy that is more effective than the comparator strategy [55]. We applied a 4.0% discount rate for the costs and 1.5% for the effects based on Dutch guidelines for health economic evaluation [56].

Strategies were compared incrementally (among each other) to identify the most cost-effective strategy. According to WHO guidelines, a strategy can be considered highly cost-effective when the ICER is < = 1 times the Gross Domestic Product (GDP) per capita and as cost-effective when the ICER < = 3 times GDP/capita [57]. The Dutch GDP/capita was €38,255 in 2013 [58], implying that ICERs below 38,255 €/QALY would be considered highly cost-effective in the Netherlands. Graphically, all strategies are depicted on the cost-effectiveness frontier (Text E in S1 File). We also assessed the effect of treatment on the HCV-RNA prevalence over time.

### Sensitivity and uncertainty analyses

We performed one-way deterministic sensitivity analyses to assess the impact of certain model parameters on the ICER in the declining epidemic. The following sensitivity analyses were done: fibrosis progression 2x the base transition probability, 0.70 SVR probability for F3-F4 in the SOF/RBV strategy (instead of 0.90), 40% never screened for HCV (instead of 20%), higher and lower utility values (+0,1,+0,2 and -0,1. -0,2), 20% and 50% lower DAA costs, excluding costs specific for PWID care, and a 0% discount rate for the costs and effects. A probabilistic sensitivity analysis using 1,000 bootstraps was also performed to reflect uncertainty in the costs and utilities parameter values

### Scenario analysis

We calculated the cost-effectiveness of increased treatment uptake, by treating 100 PWID per year with dual DAA in Amsterdam (the declining epidemic); corresponding to an estimated 6% treatment uptake rate among HCV-RNA positive PWID in 2015. PWID specific treatment costs were doubled in this scenario analysis.

## Results

**Fig 2:**
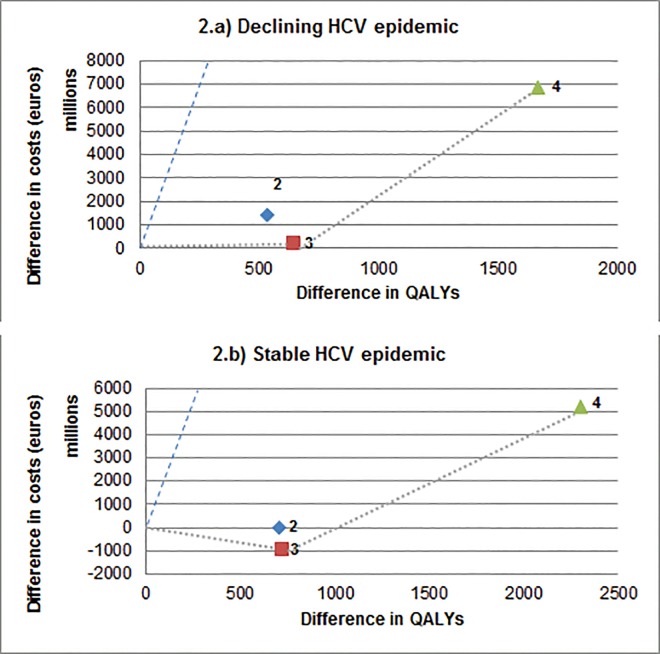
Cost-effectiveness frontier of DAA-treatment strategies among PWID compared to PegIFN/RBV^a^. Strategy 2: DAA/RBV (G2-3) & dual DAA (g1-4); 3: Dual DAA for all genotypes; 4: Dual DAA with a 3x higher treatment uptake. a The strategies that fall below the dashed blue line are strategies that fall bellow a willingness to pay threshold reflecting 1 GDP per head of the population, i.e., €38,255 for the Netherlands, and are considered highly cost-effective compared to PegIFN/RBV. However, scenarios are compared incrementally to identify the *most* cost-effective strategy. The most cost-effective strategy is shown on the “cost-effectiveness frontier”, the line that is closest to the X-axis.

Table 3 shows the cumulative discounted QALYs and costs, ICERs, and averted HCV infections. Fig 2, the cost-effectiveness frontier, illustrates the point-estimates of QALYs gained and additional costs for all treatment strategies compared to PegIFN/RBV.

**Table 3:** Cumulative discounted costs, effects, and incremental cost-effectiveness ratios of four Hepatitis C treatment strategies in a declining epidemic and a stable HCV epidemic.

	Total screening costs (Thousand €)	Total health state costs (million €)	Total treatment costs (million €)	Total costs in euro (million €)	Total number of new infections averted	Total no. of QALYs	Comparing	ICER^b^€/QALY
**Declining epidemic**								
**1. PegIFN/RBV**	3.30	23.66	2.93	26.92	2.5	16,659		
**2. DAA/RBV & dual DAA**^**a**^	3.31	23.35	4.66	28.35	2.5	17,192	2 vs. 1	**Ext.Dominated**
**3. Dual DAA**	3.29	23.50	3.32	27.14	1.7	17,300	3 vs. 1	**344**
**4. Dual DAA HU**	3.31	22.34	11.11	33.78	4.0	18,324	4 vs. 3	**4,115**
**Stable epidemic**								
**1. PegIFN/RBV**	4.78	8.24	2.77	11.49	6.7	9,802		
**3. DAA/RBV & dual DAA**^**a**^	4.73	7.12	3.90	11.49	10.0	10,508	2 vs. 1	**Dominated**
**4. Dual DAA**	4.75	7.10	3.01	10.59	11.2	10,522	3 vs. 1	**Dominant**
**4. Dual DAA HU**	4.72	6.59	9.62	16.69	30.7	12,104	4 vs. 3	**2,258**

Abbreviations: No: number; PegIFN: pegylated-interferon; RBV: ribavirin; DAA: direct-acting antiviral; ICER: incremental cost-effectiveness ratio; QALYs: quality-adjusted life years; Ext = extendedly; HU: higher uptake (3x); €: euros

^**a**^ Dual DAA therapy only for genotype 1 and 4

^b^ A strategy is ext. (extendedly) dominated when another treatment strategy is more attractive (i.e. yielding better outcomes (more QALYs)), even if that ICER falls below the willingness to pay threshold. A strategy is dominated when the costs are higher and effects are lower than the comparator strategy. A dominant strategy is better (yields more QALYs) and cheaper than the comparator strategy.

### Base case, declining epidemic

Treatment with dual DAAs was the most cost-effective strategy (strategy 3) and is highly cost-effective (ICER = 344 €/QALY) (Table 3). As illustrated in Fig 2A, all DAA strategies fall below the willingness to pay threshold (WTP) of a highly cost-effective strategy (i.e., 38,255 €/QALY). This means that when assessed on their own as compared to PegIFN/RBV, all strategies would be highly cost-effective; however when compared with each other, dual DAA therapy is most cost-effective. A small number of new infections were averted in all treatment scenarios (i.e., 2 in scenario 3) (Table 3). Over a 15-year period, dual DAA led to a 13% and 5% reduction in chronic HCV prevalence for G1-4 and G2-3 while treating 45 PWID annually (i.e., 3x higher uptake) with dual DAAs (strategy 4) led to a 19% and 8% reduction, respectively (Fig A in S1 File).

### Impact of the type of epidemic

Treatment with dual DAA in a stable epidemic was the dominant strategy and led to cost-savings (Table 3 and Fig 2B). Eleven new infections were averted with dual DAA over the modeled period. Over a 15-year period, dual DAA led to a 5% and 3% reduction in chronic HCV prevalence for G1-4 and G2-3 while treating 45 PWID annually with dual DAAs led to a 17% and 7% reduction, respectively (Fig A in S1 File).

### Sensitivity and uncertainty analyses

**Fig 3:**
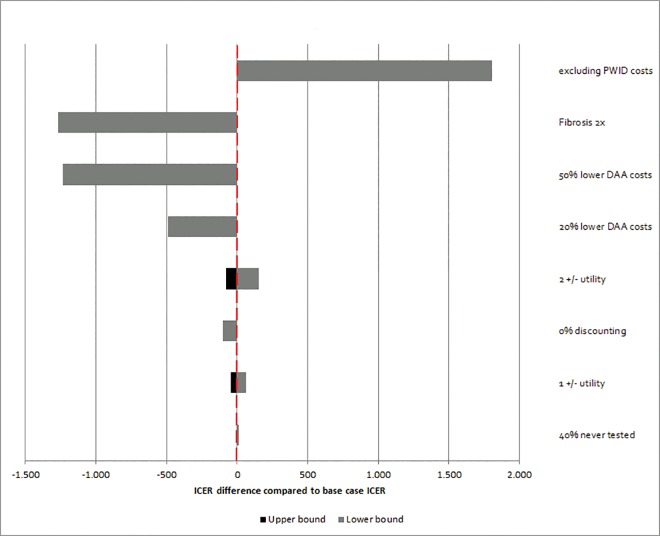
Tornado diagrams illustrating deterministic sensitivity analyses of dual DAA compared to PegIFN/RBV in the declining epidemic (Amsterdam, The Netherlands). Abbreviations: DAA: direct-acting antiviral. The red dashed line represents the base case ICER (ICER = 344 €/QALY). For the deterministic sensitivity analyses of the utilities: the grey bars represent a lower utility than the base case; the black bars represent a higher utility than the base case. Fibrosis 2x: means a fibrosis progression two times that of the base case scenario.

Dual DAA therapy remained highly cost-effective throughout the deterministic sensitivity analyses and ICERs were comparable to the main analysis. Although when DAA costs are 20% or 50% lower, and when fibrosis progression is twice that of the baseline scenario, this strategy becomes cost-saving (Fig 3). On the other hand, when specific PWID treatment costs were excluded from the analysis, the ICER for dual DAA increased to 2,150 €/QALY. The probability of dual DAA therapy being cost-effective was 100% at a WTP of one time GDP/capita. Therefore, no cost-effectiveness acceptability curves were plotted.

### Scenario analysis

When 100 PWID were treated annually in the declining epidemic, dual DAA remained highly cost-effective when compared to PegIFN/RBV (ICER = 4,192 €/QALY). This scenario led to a 23% and 6%, reduction of chronic HCV prevalence over a 15-year period for G1-4 and G2-3, respectively; though in 2029, chronic HCV prevalence was still estimated at 18% for G1-4.

## Discussion

We determined the cost-effectiveness of four HCV treatment strategies among PWID in a stable and a declining HCV epidemic. This study showed that treatment with DAA-containing regimens for PWID is a highly cost-effective intervention, irrespective of the type of epidemic. Although dual DAA therapy was most cost-effective, the two other DAA-treatment strategies fell also below the WTP. Sensitivity analyses showed that our ICERs were very robust. These analyses provide economic support for the treatment of PWID with DAAs.

Our results are in line with other cost-effectiveness studies with sofosbuvir-containing regimens among chronically HCV-infected individuals [59–62]. Hagan et al. reported that dual DAA (SOF/simeprevir (SOF/SMR)) resulted in both better outcomes and less costs compared to SOF/RBV [60]. Similarly, a cost-effectiveness study in Germany showed that SOF/SMV is both more effective and cheaper than SOF/RBV [63]. The lower costs and higher QALYs accrued in these studies and our present study with dual DAA can be explained by the shorter treatment durations and higher efficacy, which result in lower healthcare utilization costs and more prevention of liver-related morbidity (e.g., HCC). In a cost-effectiveness study of DAAs among PWID, Hellard et al. showed that in Australia, early treatment (from F0) and late treatment (from F2) yielded an ICER of 10,272 and 5,078 Australian dollars, respectively, compared to no treatment [16]. However, this study did not capture the benefits of reduced transmission (treatment as prevention) and therefore, ICERs might be even more favorable in reality than those reported. Anothers cost-effectiveness study also showed that treating PWID is cost effective in the UK; especially among PWID with moderate fibrosis at a 40% baseline chronic HCV prevalence [64]. Our low ICERs might be a result of incorporating a healthcare model specific for PWID in our analysis, as sensitivity analysis without these costs resulted in a higher ICER. Also, we included fibrosis progression and a clearance rate specific for HIV/HCV-coinfected which is usually not explicitly incorporated in cost-effectiveness analyses for HCV treatment [16, 63–65]. As HIV/HCV-coinfection leads to faster HCV disease progression [31] and our sensitivity analysis showed that this parameter (faster fibrosis progression) had the biggest impact on the ICER, excluding these HIV-related parameters in the model would result in less favorable ICERs. Furthermore, in the stable epidemic dual DAA led to cost-savings. This might be a result of preventing HCV transmission (population benefit) compared to the declining epidemic with few new infections among PWID. Healthcare costs were also higher in the declining than the stable epidemic, which is probably a result of the younger PWID population in the stable epidemic that has not progressed to advance fibrosis stages (which incur higher costs than early fibrosis stages). Hence more liver-related morbidity can be prevented in the stable epidemic compared to the declining epidemic.

Although DAA-containing treatments are cost-effective, treating only 15 PWID annually will probably not contain the epidemic in an on-going transmission setting (stable epidemic) as only a slight decrease in chronic HCV prevalence was observed with dual DAA in this epidemiological setting. When treatment is scaled-up (i.e., 45 PWID treated annually) more new infections were averted (31 vs. 11 with baseline uptake) and HCV RNA prevalence decreased 17% for G1-4. Martin et al. also showed that current treatment uptake in England was unlikely to achieve observable reductions in HCV prevalence, while scaling up treatment (to 26/1000 annually) could lead to a substantial reduction [66]. However, the absolute number needed to treat in other settings may differ from our analysis and may depend on the population size. In the declining epidemic, a higher reduction of chronic HCV prevalence is observed even when treating 15 PWID annually compared to the stable epidemic (13% vs. 5% reduction for G1-4 in the declining and stable epidemic, respectively). This is probably a result of HCV-treatment and mortality in this ageing PWID population. Although treating 100 PWID annually led to a substantial reduction of HCV RNA prevalence (23% for G1-4) and was also highly cost-effective. Nevertheless, still after 15 years, HCV RNA prevalence was 18% for G1-4, suggesting that to eliminate HCV among PWID in Amsterdam, treatment should be scale-up even further and those who abstain from screening or HRP should be identified and actively approached.

Our analyses have several limitations. First, SVR for DAAs are mainly based on clinical trials with relatively small sample sizes. However, a recent real-life study showed comparable SVR rates as those observed in clinical trials [67]. Second, we assumed that there was no difference in SVR for SOF/RBV for G2-3 with F3-F4 as only limited trial data was available at the time the model was built, although sensitivity analysis showed similar results. If SVRs were overestimated in our study, this could have led to more favorable ICERs compared to PegIFN/RBV. On the other hand, we might have overestimated the costs of (pre- and post-)DAA treatment monitoring as we made conservative assumptions on frequency of laboratory/diagnostic tests and clinical visits. In real life, less healthcare utilization might be feasible when no PegIFN and/or RBV are given, as these medications cause significantly more side effects than DAAs [8]. Costs due to adverse events for DAAs were assumed to be half of cost accrued with PegIFN/RBV, but we believe those costs might be even lower in a real-life setting. Also, our analysis used list prices for DAAs, while actual costs may be lower after price negotiations. Lower costs of DAA treatment would make DAAs even more favorable than PegIFN/RBV as shown in our sensitivity analyses. Furthermore, for simplicity, we did not account for the percentage of those ineligible for or intolerant to PegIFN. Also, we assumed that HRP, such as low-threshold methadone programs where PWID could be screened for HCV, were in place. Although this is true for Amsterdam, our results from the stable epidemic might not be generalizable to countries without wide coverage of such programs, as only screening costs were taken into account. Furthermore, for the stable HCV epidemic analysis, key parameters from the model may not be appropriate for countries with a stable epidemic (e.g., screening coverage). Therefore, caution must be taken when extrapolating these results. Last but not least, real-life DAA studies among PWID with large sample sizes are necessary to confirm our assumptions on SVR in this population. Furthermore, other treatment models, such as HCV treatment fully integrated into methadone maintenance programs or supervised injecting facilities [68], might reduce treatment costs compared to our integrated treatment setting. Lower healthcare utilization costs is likely to be the case with DAAs as we believe that less health-provider support might be necessary as fewer significant side effects can be expected. Future research should evaluate the cost-effectiveness of different treatment models for PWID with DAA-containing regimens.

There are also several strengths in this study. First, we used an individual-based transmission model which took re-infections into account and thus the population benefit of HCV treatment; particularly important for countries with on-going HCV transmission. If re-infections are not taken into account, the burden of HCV might be underestimated. Second, our study is mostly based on observed data from Amsterdam on incidence, prevalence, mortality, HCV treatment uptake, and real-world DAA prices. Also, we took screening and a treatment setting specific for PWID into account, which is usually not included in recent cost-effectiveness studies for DAA-containing regimens [16, 59–62, 64]. Third, we analyzed different HCV treatment scenarios among PWID to depict the possible choices of treatment based on current Dutch guidelines.

In conclusion, DAA-containing regimens are highly cost-effective among PWID, irrespective of the type of HCV epidemic. Given the current evidence, dual DAA therapy should be considered the standard recommended HCV treatment, not only because of its higher efficacy but also the lower net costs compared to other DAA regimens [60, 63]. Also, based on the economic and population benefits of scaling-up treatment, stronger efforts are needed to achieve higher uptake rates among PWID.

## Supporting Information

S1 File Supplementary tables & graphs. (DOCX)

## Abbreviations

PWID: People who inject drugs
HCV: Hepatitis C virus
DAAs: Direct-acting antivirals
QALYs: Quality-adjusted life years
PegIFN: Pegylated-interferon
RBV: Ribavirin
SVR: Sustained virologic response
ACS: Amsterdam Cohort Studies
ICER: Incremental cost-effectiveness ratio
DC: Decompensated cirrhosis
HCC: Hepatocellular carcinoma
