# Supplementary material for: Cost-Effectiveness of Hepatitis C Treatment for People Who Inject Drugs and the Impact of the Type of Epidemic; Extrapolating from Amsterdam, the Netherlands
Source: PLoS One. 2016 Oct 6;11(10):e0163488. doi: 10.1371/journal.pone.0163488 (PMC5053429; doi:10.1371/journal.pone.0163488)
Supplement: S1 File — (DOCX) [file pone.0163488.s001.docx]

| **Supplementary Tables & Figures**  **Supplementary text methods:**  **A Model expansion**  Genotype: we categorized genotype into two groups, genotype 1-4 or genotype 2-3. Studies have shown evidence to suggest at least partial HCV-related immunity ^[1, 2]^ and although the strength of this immunity effect remains unclear, we assumed that PWID who cleared the virus had a 20% lower probability to become re-infected with HCV with the same genotype, but were equally susceptible to get infected with another genotype group.  **B Natural history**  Transition probabilities are based on original research or previous modeling studies. HCV clearance has been shown to be dependent on HIV-coinfection status^[3]^.Therefore, in our model, HCV/HIV-coinfected PWID had a lower HCV clearance rate (17% vs. 25% among HCV-monoinfected ^[4]^); this percentage was based on two prospective longitudinal studies reporting a clearance rate of 15% and 20% ^[3, 5]^ . Furthermore, based on a systematic review of longitudinal studies, females have a higher probability to clear HCV^[6]^. Fibrosis progression based on the METAVIR scoring system was dependent on sex and age group^[7]^ based on an empirically-calibrated model from the US, and assumed to be twice as fast among HCV/HIV-coinfection status based on a meta-analyses^[8]^. An individual with cirrhosis (METAVIR: F4) could develop decompensated cirrhosis (DC) or hepatocellular carcinoma (HCC), which is dependent on HIV-coinfection status^[9]^ (by a factor of 1.5^[9]^). The HCC- and DC-related mortality rates do not depend on HIV status. For simplicity, we assumed that during treatment, individuals could not transmit HCV to other PWID. After achieving SVR individuals remained in their fibrosis stage, without progressing to other fibrosis stages^[10, 11]^. After a re-infection, individuals could progress to another fibrosis health state.  **C Treatment uptake**  We assumed that 15 PWID were treated annually at the PHSA irrespective of their fibrosis stage. This was based on data from the DUTCH-C project where on average 10 PWID were treated per year until 2013; of whom around 7% were HCV/HIV-coinfected. At the beginning, only HCV-monoinfected PWID were treated. Later, treatment was extended to HCV/HIV-coinfected PWID. Based on expert medical opinion, few PWID, and usually more stable (ex-)PWID, were treated outside the DUTCH-C project from 2005 until 2013, therefore we assumed that an additional 5 PWID could be treated. Though, in our model, all 15 PWID were treated at the PHSA as we included a model of care specific for PWID. We assumed the same treatment uptake in the stable epidemic scenario; however, in contrast to the declining epidemic, the proportion of HCV/HIV-coinfected who were treated was proportional to the HIV prevalence among HCV-infected PWID as we assume this to be a more realistic scenario given that DAAs have been shown to be well tolerated by HCV/HIV-coinfected individuals^[12]^. Furthermore, treatment with PegIFN/RBV started in 2005 in the declining epidemic until 2014 (unless the strategy from 2015 onwards was PegIFN/RBV), based on the situation in Amsterdam at that time. Starting date of HCV treatment in the stable epidemic was 2015.    **D Treatment options SVR sources**   1. Treatment with PegIFN with RBV (PegIFN/RBV): SVR rates were dependent on HIV status and fibrosis stage^[13-15]^. 2. SOF/RBV for genotype 2-3 and dual DAA for genotype 1-4: SVR rates for genotypes 2 are based on averaged results from the FISSION, POSITRON, and VALENCE trial ^[16-18]^. The latter trial was used to obtain the SVR for genotype 3. Dual DAA therapy SVRs are based on averaged results from trials with daclatasvir combined with sofosbuvir^[19, 20]^. 3. Dual DAA therapy for all genotypes: SVRs were the same as in scenario 3 for all genotypes.   For simplicity, no fibrosis to moderate fibrosis (F0-F2) and severe fibrosis and cirrhosis are grouped together (F3-F4). Therefore, treatment duration with SOF/RBV for individuals with cirrhosis and genotype 2 is underestimated (12 weeks instead of 16)^[21]^, although very few PWID are infected with this genotype. Only 4% of HCV-infected participants from the Amsterdam Cohort Study had a genotype 2 infection during 2000-2004^[22]^.s  **E Analyses & model output**  A strategy can be (highly) cost-effective, dominant, dominated, or extendedly dominated. Strategies are ruled out by either dominance or extended dominance. Strategies with higher costs and lower effects than a comparator strategy are considered to be dominated and are no longer used as a comparator strategy for the next strategies. Strategies are extendedly dominated when another strategy has lower costs and more QALYs; even if the extendedly dominated strategy has an ICER that falls below the willingness to pay threshold. A strategy that is more effective (more QALYs) is compared to a previous strategy that has not been previously dominated or extendedly dominated. We used a 15 year time horizon as we believe that it is sufficient to capture the effects of treatment on healthcare-related costs in this ageing PWID population in the Netherlands^[23]^.  **Model output:**  From 2015 onwards, the model annually estimated the following for each strategy:   1. The number of PWID screened, the number of HCV-antibody-positive (HCVab+) among those screened, and HCV-RNA-positive among HCVab+; 2. Number of person months during treatment per genotype group and fibrosis stage; 3. The number of person months per fibrosis stage and genotype group: during treatment among HCV-RNA-positive PWID (treatment-naïve), unsuccessfully treated, and among those who achieved SVR; 4. The number of person months with HCC or DC and the number of deceased PWID; 5. HCV-antibody and -RNA incidence and prevalence by genotype group.   **Supplementary Table A:** Overview of HCV treatment scenarios in the stable and the declining HCV epidemic among PWID.   \| **Scenario:** \| **Genotype group** \| **Weeks** \| **Genotype group** \| **Weeks** \| \| --- \| --- \| --- \| --- \| --- \| \|  \| 1-4 \|  \| 2-3 \|  \| \| 1 \| PegIFN/RBV \| 48 \| PegIFN/RBV \| 24 \| \| 2 \| Dual DAA \| 12 \| DAA/RBV \| 22* \| \| 3 & 4 \| Dual DAA \| 12 \| Dual DAA \| 12 \|   * weighted average = 24 weeks of treatment for genotype 3 and 12 weeks for genotype 2; As only 20% ^[22]^ in this genotype group is infected with genotype 2, the weighted number of weeks of treatment is in between 20 and 24 weeks. We averaged the latter two, and rounded it to 22 weeks of treatment.  **Supplementary Table B: Costs of HCV treatment & screening**   \|  \| **Costs** \| **year** \| **source ^g^** \| **Costs in 2014^j^** \| **PegIFN PegIFN** \| \| \| \| \| **SOF/RBV Dual DAA** \| \| \| \| \| \| \| \| \| \| \| --- \| --- \| --- \| --- \| --- \| --- \| --- \| --- \| --- \| --- \| --- \| --- \| --- \| --- \| --- \| --- \| --- \| --- \| --- \| --- \| \|  \|  \|  \|  \|  \| **G1-4 G2-3** \| \| \| \| \|  \| \| \| \| \| \| \| \| \| \| \| **Staff costs (includes overhead costs)** \| \| Medical specialist consultation (hour) and medical coordinator \| 103 \| 2009 \| ^[24]^ \| 113.2 \| 1,188.7 \| \| \| 849.1 \| 792.5 \| \| \| \| \| 792.5 \| \| \| \| \| Nurse consultation (hour) \|  \| 2014 \| PHSA \| 62.2 \| 11,944.3 \| \| \| 5,972.2 \| 2,986.1 \| \| \| \| \| 1,493.0 \| \| \| \| \| Psychiatric consultation (hour) \| 103 \| 2009 \| ^[24]^ \| 113.2 \| 113.2 \| \| \| 113.2 \| 113.2 \| \| \| \| \| 113.2 \| \| \| \| \| Cardiologist/Pulmonologist (hour) ^a^ \| 103 \| 2009 \|  \| 56.6 \| 56.6 \| \| \| 56.6 \| 56.6 \| \| \| \| \| 56.6 \| \| \| \| \| HIV specialist (hour) ^b^ \| 103 \| 2009 \|  \| 7.9 \| 15.8 \| \| \| 15.8 \| 15.8 \| \| \| \| \| 15.8 \| \| \| \| \|  \|  \|  \|  \|  \|  \| \| \| \| \| \| \| \| \| \| \| \| \| \| \| \| **HCV screening ^c^** \|  \|  \|  \|  \| \| HCV antibodies \|  \| 2014 \| PHSA \| 11.4 \| \| HCV RNA + genotype \|  \| 2014 \| AMC \| 222.8 \| \|  \|  \|  \|  \|  \| \| **Routine laboratory tests** \|  \|  \|  \|  \|  \| \| \| \| \| 194.7 \| \| \| \|  \| 97.35 \| \| \| \| \| Hemoglobin \|  \| 2014 \| 70702 \| 1.7 \|  \|  \| \| \| \| \| \| \| \| \|  \| \| \| \| \| \| Leucocyte \|  \| 2014 \| 70741 \| 1.7 \|  \|  \| \| \| \| \| \| \| \| \|  \| \| \| \| \| \| Leucocyte differential \|  \| 2014 \| AMC \| 2.3 \|  \|  \| \| \| \| \| \| \| \| \|  \| \| \| \| \| \| Thrombocytes \|  \| 2014 \| 70715 \| 1.7 \|  \|  \| \| \| \| \| \| \| \| \|  \| \| \| \| \| \| ALAT \|  \| 2014 \| 74891 \| 2.1 \|  \|  \| \| \| \| \| \| \| \| \|  \| \| \| \| \| \| ASAT \|  \| 2014 \| 74489 \| 1.9 \|  \|  \| \| \| \| \| \| \| \| \|  \| \| \| \| \| \| Alkaline phosphatase \|  \| 2014 \| 74896 \| 2 \|  \|  \| \| \| \| \| \| \| \| \|  \| \| \| \| \| \| Gamma - GT \|  \| 2014 \| 72417 \| 1.9 \|  \|  \| \| \| \| \| \| \| \| \|  \| \| \| \| \| \| Glucose \|  \| 2014 \| 70402 \| 1.8 \|  \|  \| \| \| \| \| \| \| \| \|  \| \| \| \| \| \| HbA1c (if glucose is high) ^d^ \|  \| 2014 \| 74065 \| 2.4 \|  \|  \| \| \| \| \| \| \| \| \|  \| \| \| \| \| \|  \| \|  \|  \|  \|  \|  \| \| \| \| \| \| \| \| \|  \| \| \| \| \| \| **Screening diagnostic tests** \|  \|  \|  \|  \|  \| \| \| \| \| \| 32.7 \| \| \| \| 32.7 \| \| \| \| \| \| PTT \|  \| 2014 \| 70707 \| 4.1 \|  \| \| \| \| \| \|  \| \| \| \|  \| \| \| \| \| \| APTT \|  \| 2014 \| 77371 \| 3.3 \|  \| \| \| \| \| \|  \| \| \| \|  \| \| \| \| \| \| AT III \|  \| 2014 \| AMC \| 13.5 \|  \| \| \| \| \| \|  \| \| \| \|  \| \| \| \| \| \| Albumin \|  \| 2014 \| 74802 \| 1.6 \|  \| \| \| \| \| \|  \| \| \| \|  \| \| \| \| \| \| Creatine \|  \| 2014 \| 70128 \| 1.8 \|  \| \| \| \| \| \|  \| \| \| \|  \| \| \| \| \| \| ANA \|  \| 2014 \| 70693 \| 8.4 \|  \| \| \| \| \| \|  \| \| \| \|  \| \| \| \| \| \|  \| \| \|  \|  \|  \| \| \| \| \| \| \| \| \| \|  \| \| \| \| \|  \|  \|  \| \| **RNA concentration** \|  \|  \|  \| \|  \| \| \| \|  \| \| \| \|  \| \|  \| \| \| \| \| \| Qualitative & quantitative \|  \| 2014 \| AMC \| 126.8 \|  \| \| \| \| \| \| 760.7 \| \| \| \| 760.7 \| \| \| \| \| \|  \|  \|  \|  \|  \|  \| \| \| \| \| \|  \| \| \| \|  \| \| \| \| \| \| **Endocrinology** \|  \|  \|  \|  \|  \| \| \| \| \| \|  \| \| \| \|  \| \| \| \| \| \| TSH \|  \| 2014 \| 72573 \| 6.7 \|  \| \| \| \| \| \| 20.1 \| \| \| \| 20.1 \| \| \| \| \| \|  \|  \|  \|  \|  \|  \| \| \| \| \| \|  \| \| \| \|  \| \| \| \| \| \| **Radiology** \|  \|  \|  \|  \|  \| \| \| \| \| \| 966.3 \| \| \| \| 966.3 \| \| \| \| \| \| Echo \|  \| 2014 \| AMC \| 58.7 \|  \| \| \| \| \| \|  \| \| \| \|  \| \| \| \| \| \| X ray thorax \|  \| 2014 \| 85070 \| 44.2 \|  \| \| \| \| \| \|  \| \| \| \|  \| \| \| \| \| \| FibroScan \| 120 \| 2012 \| AMC \| 126.1 \|  \| \| \| \| \| \|  \| \| \| \|  \| \| \| \| \| \| MRI liver ^e^ \| 118.51 \| 2014 \| AMC \|  \|  \| \| \| \| \| \|  \| \| \| \|  \| \| \| \| \| \| Endoscopy \|  \| 2014 \| 34620 \| 210.8 \|  \| \| \| \| \| \|  \| \| \| \|  \| \| \| \| \| \|  \|  \|  \|  \|  \|  \| \| \| \| \| \|  \| \| \| \|  \| \| \| \| \| \| **ECG ^f^** \|  \| 2014 \| 39757 \| 43.4 \|  \| \| \| \| \| \| \| 43.4 \| \| \| 43.4 \| \| \| \| \| \| **Medication costs ^h,i^** \|  \|  \| 27,^[25]^ \| \| 16,393 \| \| \| 12,291 \| \| \| \| 99,485 \| \| \| 79,195 \| \| \| \| \| \| **Side effects DAA** \|  \|  \| ^[26]^ \| 1009.3 \|  \| \| \| \| \| \|  \| \| \| \| \| \|  \| \| \| \| **TOTAL TREATMENT COSTS** \|  \|  \|  \| \| **29,712** \| \| **19,298** \| \| \| \| **106,476** \| \| \| \| \| **84,216** \| \| \| \|   Abbreviations: PHSA: Public Health Service of Amsterdam; AMC: Amsterdam Medical Center; PTT: partial thromboplastic time; APTT: activated PTT;  AT: antithrombin; ANA: antinuclear antibody; TSH: thyroid stimulating hormone; ECG: electrocardiogram; G: genotype; SOF: sofosbuvir |  |  |  |  |  |  |  |  |  |  |  |
| --- | --- | --- | --- | --- | --- | --- | --- | --- | --- | --- | --- | --- | --- | --- | --- | --- | --- | --- | --- | --- | --- | --- | --- | --- | --- | --- | --- | --- | --- | --- | --- | --- | --- | --- | --- | --- | --- | --- | --- | --- | --- | --- | --- | --- | --- | --- | --- | --- | --- | --- | --- | --- | --- | --- | --- | --- | --- | --- | --- | --- | --- | --- | --- | --- | --- | --- | --- | --- | --- | --- | --- | --- | --- | --- | --- | --- | --- | --- | --- | --- | --- | --- | --- | --- | --- | --- | --- | --- | --- | --- | --- | --- | --- | --- | --- | --- | --- | --- | --- | --- | --- | --- | --- | --- | --- | --- | --- | --- | --- | --- | --- | --- | --- | --- | --- | --- | --- | --- | --- | --- | --- | --- | --- | --- | --- | --- | --- | --- | --- | --- | --- | --- | --- | --- | --- | --- | --- | --- | --- | --- | --- | --- | --- | --- | --- | --- | --- | --- | --- | --- | --- | --- | --- | --- | --- | --- | --- | --- | --- | --- | --- | --- | --- | --- | --- | --- | --- | --- | --- | --- | --- | --- | --- | --- | --- | --- | --- | --- | --- | --- | --- | --- | --- | --- | --- | --- | --- | --- | --- | --- | --- | --- | --- | --- | --- | --- | --- | --- | --- | --- | --- | --- | --- | --- | --- | --- | --- | --- | --- | --- | --- | --- | --- | --- | --- | --- | --- | --- | --- | --- | --- | --- | --- | --- | --- | --- | --- | --- | --- | --- | --- | --- | --- | --- | --- | --- | --- | --- | --- | --- | --- | --- | --- | --- | --- | --- | --- | --- | --- | --- | --- | --- | --- | --- | --- | --- | --- | --- | --- | --- | --- | --- | --- | --- | --- | --- | --- | --- | --- | --- | --- | --- | --- | --- | --- | --- | --- | --- | --- | --- | --- | --- | --- | --- | --- | --- | --- | --- | --- | --- | --- | --- | --- | --- | --- | --- | --- | --- | --- | --- | --- | --- | --- | --- | --- | --- | --- | --- | --- | --- | --- | --- | --- | --- | --- | --- | --- | --- | --- | --- | --- | --- | --- | --- | --- | --- | --- | --- | --- | --- | --- | --- | --- | --- | --- | --- | --- | --- | --- | --- | --- | --- | --- | --- | --- | --- | --- | --- | --- | --- | --- | --- | --- | --- | --- | --- | --- | --- | --- | --- | --- | --- | --- | --- | --- | --- | --- | --- | --- | --- | --- | --- | --- | --- | --- | --- | --- | --- | --- | --- | --- | --- | --- | --- | --- | --- | --- | --- | --- | --- | --- | --- | --- | --- | --- | --- | --- | --- | --- | --- | --- | --- | --- | --- | --- | --- | --- | --- | --- | --- | --- | --- | --- | --- | --- | --- | --- | --- | --- | --- | --- | --- | --- | --- | --- | --- | --- | --- | --- | --- | --- | --- | --- | --- | --- | --- | --- | --- | --- | --- | --- | --- | --- | --- | --- | --- | --- | --- | --- | --- | --- | --- | --- | --- | --- | --- | --- | --- | --- | --- | --- | --- | --- | --- | --- | --- | --- | --- | --- | --- | --- | --- | --- | --- | --- | --- | --- | --- | --- | --- | --- | --- | --- | --- | --- | --- | --- | --- | --- | --- | --- | --- | --- | --- | --- | --- | --- | --- | --- | --- | --- | --- | --- | --- | --- | --- | --- | --- | --- | --- | --- | --- | --- | --- | --- | --- | --- | --- | --- | --- | --- | --- | --- | --- | --- | --- | --- | --- | --- | --- | --- | --- | --- | --- | --- | --- | --- | --- | --- | --- | --- | --- | --- | --- | --- | --- | --- | --- | --- | --- | --- | --- | --- | --- | --- | --- | --- | --- | --- | --- | --- | --- | --- | --- | --- | --- | --- | --- | --- | --- | --- | --- | --- | --- | --- | --- | --- | --- | --- | --- | --- | --- | --- | --- | --- | --- | --- | --- | --- | --- | --- | --- | --- | --- | --- | --- | --- | --- | --- | --- | --- | --- | --- | --- | --- | --- | --- | --- | --- | --- | --- | --- | --- | --- | --- | --- | --- | --- | --- | --- | --- | --- | --- | --- | --- | --- | --- | --- | --- | --- | --- | --- | --- | --- | --- | --- | --- | --- | --- | --- | --- | --- | --- | --- | --- | --- | --- | --- | --- | --- | --- | --- | --- | --- | --- | --- | --- | --- | --- | --- | --- | --- | --- | --- | --- | --- | --- | --- | --- | --- | --- | --- | --- | --- | --- | --- | --- | --- | --- | --- | --- | --- | --- | --- | --- | --- | --- | --- | --- | --- | --- | --- | --- | --- | --- | --- | --- | --- | --- | --- | --- | --- | --- | --- | --- | --- | --- | --- | --- | --- | --- | --- | --- | --- | --- | --- | --- | --- | --- | --- | --- | --- | --- | --- | --- | --- | --- | --- | --- | --- | --- | --- | --- | --- | --- | --- | --- | --- | --- | --- | --- | --- | --- | --- | --- | --- | --- | --- | --- | --- | --- | --- | --- | --- | --- | --- | --- | --- | --- | --- | --- | --- | --- | --- | --- | --- | --- | --- | --- | --- | --- | --- | --- | --- | --- | --- | --- | --- | --- | --- | --- | --- | --- | --- | --- | --- | --- | --- | --- | --- | --- | --- | --- | --- | --- | --- | --- | --- | --- | --- | --- | --- | --- | --- | --- | --- | --- | --- | --- | --- | --- | --- | --- | --- | --- | --- | --- | --- | --- | --- | --- | --- | --- | --- | --- | --- | --- | --- | --- | --- | --- | --- | --- | --- | --- | --- | --- | --- | --- | --- | --- | --- | --- | --- | --- | --- | --- | --- | --- | --- | --- | --- | --- | --- | --- | --- | --- | --- | --- | --- | --- | --- | --- | --- | --- | --- | --- | --- | --- | --- | --- | --- | --- | --- | --- | --- | --- | --- | --- | --- | --- | --- | --- | --- | --- | --- | --- | --- | --- | --- | --- | --- | --- | --- | --- | --- | --- | --- | --- | --- | --- | --- | --- | --- | --- | --- | --- | --- | --- | --- | --- | --- | --- | --- | --- | --- | --- | --- | --- | --- | --- | --- | --- | --- | --- | --- | --- | --- | --- | --- | --- | --- | --- | --- | --- | --- | --- | --- | --- | --- | --- | --- | --- | --- | --- | --- | --- | --- | --- |

| ^a^ For 50% of PWID based on expert opinion (incorporated in total costs in 2014) |
| --- |
| ^b^ 7% of PWID (based on the declining epidemic model). Total HIV specialist costs based on two visits (pre- and post-HCV treatment)  ^c^ Included in screening costs only  ^d^ Assumed 30% high glucose level ; 30% * 7,9 = 2,37 euros  ^e^ Not included in the treatment costs  ^f^ Standard for PWID  ^g^ Numbers shown in this column are derived from the Dutch Health Authority (NZA)  ^h^ PegIFN/RBV side effects costs already included in the mean total costs; costs for PegIFN-containing treatment were indexed to 2014 euros  ^i^ RBV included in total costs when applicable  j The column “costs in 2014” denote the cost per test or medical consultation while costs depicted under each treatment strategy incorporate total costs of  these services/tests during the whole (pre-, during and post-HCV) treatment period, when applicable.  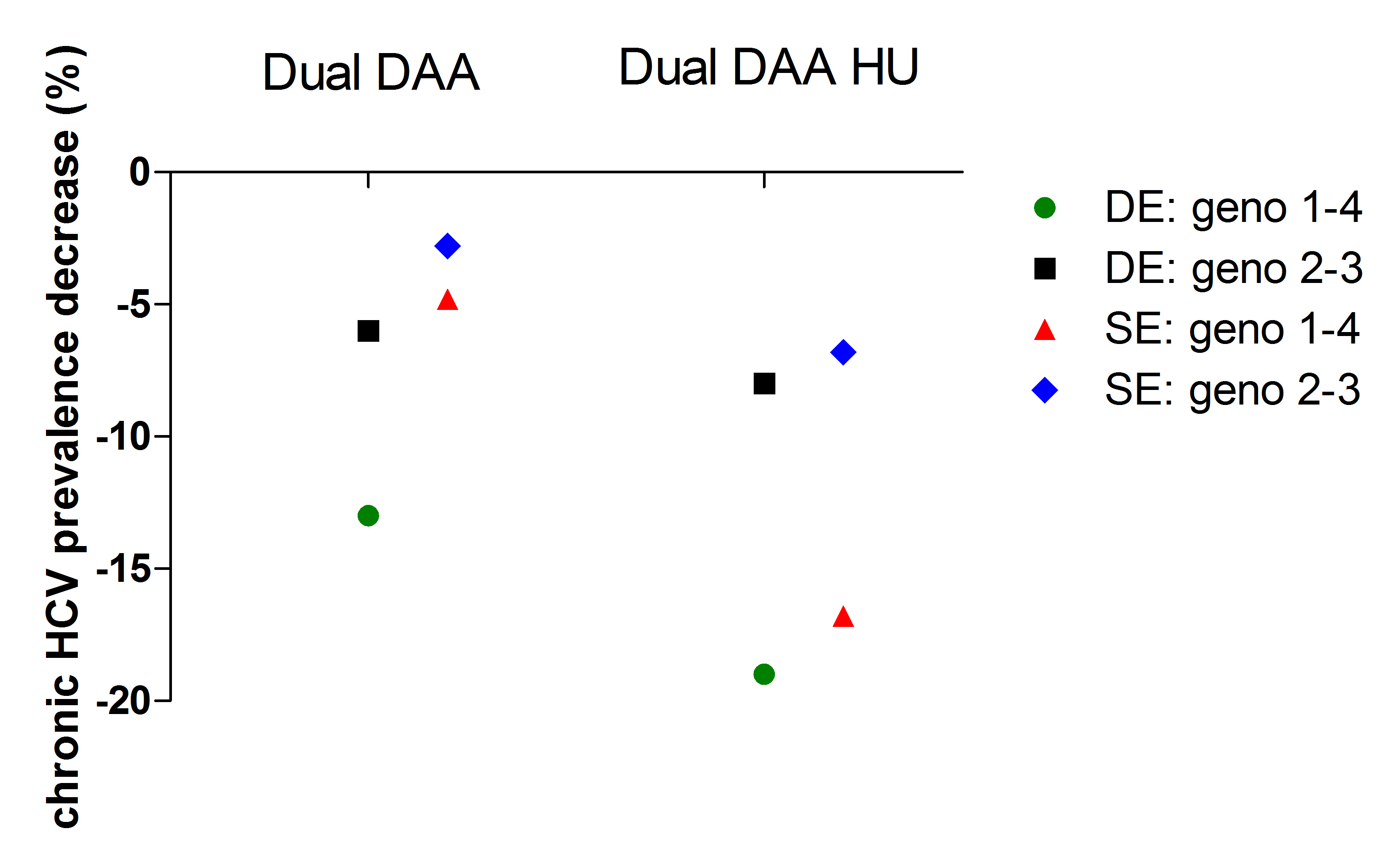  **Supplementary figure A: Chronic HCV prevalence decrease in the declining and the stable epidemic over a 15-year period**  Abbreviations: DAA: direct-acting antiviral; HU: higher uptake (3x higher uptake than the base case); geno: genotype;  DE= declining epidemic; SE= stable epidemic |

Reference List

1. Grady BP, Schinkel J, Thomas XV, Dalgard O. Hepatitis C virus reinfection following treatment among people who use drugs**.** *Clin Infect Dis* 2013**; 57 Suppl 2**:S105-S110.

2. Grebely J, Conway B, Raffa JD, Lai C, Krajden M, Tyndall MW. Hepatitis C virus reinfection in injection drug users**.** *Hepatology* 2006**; 44**:1139-1145.

3. Thomson EC, Fleming VM, Main J, Klenerman P, Weber J, Eliahoo J*, et al.* Predicting spontaneous clearance of acute hepatitis C virus in a large cohort of HIV-1-infected men**.** *Gut* 2011**; 60**:837-845.

4. Micallef JM, Kaldor JM, Dore GJ. Spontaneous viral clearance following acute hepatitis C infection: a systematic review of longitudinal studies**.** *J Viral Hepat* 2006**; 13**:34-41.

5. Soriano V, Mocroft A, Rockstroh J, Ledergerber B, Knysz B, Chaplinskas S*, et al.* Spontaneous viral clearance, viral load, and genotype distribution of hepatitis C virus (HCV) in HIV-infected patients with anti-HCV antibodies in Europe**.** *J Infect Dis* 2008**; 198**:1337-1344.

6. Micallef JM, Kaldor JM, Dore GJ. Spontaneous viral clearance following acute hepatitis C infection: a systematic review of longitudinal studies**.** *J Viral Hepat* 2006**; 13**:34-41.

7. Salomon JA, Weinstein MC, Hammitt JK, Goldie SJ. Empirically calibrated model of hepatitis C virus infection in the United States**.** *Am J Epidemiol* 2002**; 156**:761-773.

8. Thein HH, Yi Q, Dore GJ, Krahn MD. Natural history of hepatitis C virus infection in HIV-infected individuals and the impact of HIV in the era of highly active antiretroviral therapy: a meta-analysis**.** *AIDS* 2008**; 22**:1979-1991.

9. Matser A, Urbanus A, Geskus R, Kretzschmar M, Xiridou M, Buster M*, et al.* The effect of hepatitis C treatment and human immunodeficiency virus (HIV) co-infection on the disease burden of hepatitis C among injecting drug users in Amsterdam**.** *Addiction* 2012**; 107**:614-623.

10. Poynard T, Moussalli J, Munteanu M, Thabut D, Lebray P, Rudler M*, et al.* Slow regression of liver fibrosis presumed by repeated biomarkers after virological cure in patients with chronic hepatitis C**.** *J Hepatol* 2013**; 59**:675-683.

11. Zator ZA, Chung RT. After the cure: management of HCV after achievement of SVR**.** *Curr HIV/AIDS Rep* 2013**; 10**:428-435.

12. Molina JM, Orkin C, Iser DM, Zamora FX, Nelson M, Stephan C*, et al.* Sofosbuvir plus ribavirin for treatment of hepatitis C virus in patients co-infected with HIV (PHOTON-2): a multicentre, open-label, non-randomised, phase 3 study**.** *Lancet* 2015**; 385**:1098-1106.

13. Jacobson IM, McHutchison JG, Dusheiko G, Di Bisceglie AM, Reddy KR, Bzowej NH*, et al.* Telaprevir for previously untreated chronic hepatitis C virus infection**.** *N Engl J Med* 2011**; 364**:2405-2416.

14. Mira JA, Garcia-Rey S, Rivero A, de LS-G, I, Lopez-Cortes LF, Giron-Gonzalez JA*, et al.* Response to pegylated interferon plus ribavirin among HIV/hepatitis C virus-coinfected patients with compensated liver cirrhosis**.** *Clin Infect Dis* 2012**; 55**:1719-1726.

15. Powis J, Peltekian KM, Lee SS, Sherman M, Bain VG, Cooper C*, et al.* Exploring differences in response to treatment with peginterferon alpha 2a (40kD) and ribavirin in chronic hepatitis C between genotypes 2 and 3**.** *J Viral Hepat* 2008**; 15**:52-57.

16. Jacobson IM, Gordon SC, Kowdley KV, Yoshida EM, Rodriguez-Torres M, Sulkowski MS*, et al.* Sofosbuvir for hepatitis C genotype 2 or 3 in patients without treatment options**.** *N Engl J Med* 2013**; 368**:1867-1877.

17. Lawitz E, Mangia A, Wyles D, Rodriguez-Torres M, Hassanein T, Gordon SC*, et al.* Sofosbuvir for previously untreated chronic hepatitis C infection**.** *N Engl J Med* 2013**; 368**:1878-1887.

18. Zeuzem S, Dusheiko GM, Salupere R, Mangia A, Flisiak R, Hyland RH*, et al.* Sofosbuvir and ribavirin in HCV genotypes 2 and 3**.** *N Engl J Med* 2014**; 370**:1993-2001.

19. Wyles DL, Ruane PJ, Sulkowski MS, Dieterich D, Luetkemeyer A, Morgan TR*, et al.* Daclatasvir plus Sofosbuvir for HCV in Patients Coinfected with HIV-1**.** *N Engl J Med* 2015**; 373**:714-725.

20. Sulkowski MS, Jacobson IM, Nelson DR. Daclatasvir plus sofosbuvir for HCV infection**.** *N Engl J Med* 2014**; 370**:1560-1561.

21. *Richtsnoer behandeling hepatitis C infectie.* 2015.

22. van de Laar TJ, Langendam MW, Bruisten SM, Welp EA, Verhaest I, van Ameijden EJ*, et al.* Changes in risk behavior and dynamics of hepatitis C virus infections among young drug users in Amsterdam, the Netherlands**.** *J Med Virol* 2005**; 77**:509-518.

23. Van Santen DK, Van Der Helm JJ, Grady BP, de Vos AS, Kretzschmar ME, Stolte IG*, et al.* Temporal trends in mortality among people who use drugs compared with the general Dutch population differ by hepatitis C virus and HIV infection status**.** *AIDS* 2014.

24. Hakkaart- van Roijen L., Tan S.S, and Bouwmans C.A.M. *Handleiding voor kostenonderzoek.* 2009.

25. Medicijnkosten in Nederland. <http://www.medicijnkosten.nl/>. 4 April 2016

26. Helsper CW, Hellinga HL, van Essen GA, de Wit GA, Bonten MJ, van Erpecum KJ*, et al.* Real-life costs of hepatitis C treatment**.** *Neth J Med* 2012**; 70**:145-153.
